# Microscopical Memoranda

**Published:** 1874-06-01

**Authors:** 


### Microscopical Memoranda.

COLLATED BY LESTER CURTIS, M.D.

ORGANIZATION OF THROMBUS AFTER LIGATION. — Dr. T. Durante, giving the result of a series of observations on the microscopic appearances seen after the ligation of vessels, says: That when a single ligation is used, there is first the coagulum at and near the point of ligation, then the internal coat undergoes a kind of inflammatory change—abundant round and spindle cells replacing this coat. Among these, also, blood-vessels develop. By this means the coagulum is gradually pushed inwards and replaced until it finally disappears. The white corpuscles and fibrin of the original clot become fatty before disappearance, and the red ones flattened. The coagulum is thus a *transitory* thrombus and is replaced by the *permanent* one formed by the internal coat. This process is very limited where there is simply a ligature of a vessel, but it may be made to extend a considerable distance by simultaneously painting the vessel with iodine. Where a double ligation is used, the process is somewhat similar, with the exception that the seat of the cellular growth is not the interior, but the middle and external coats, the internal coat being destroyed. Soon, the two proliferating coats become blended into one cellular layer, and the normal structure can not be distinguished. The cellular growth in both cases ultimately develops in the usual way into connective tissue. The view which some authors take, that the organization depends on amœboid cells, is controverted by this author, and even in experiments made with vermilion he could find no confirmation of this view. Further, two modes of softening of the thrombus are distinguished, which have a very different significance. The one is the softening of the transitory thrombus, which precedes its absorption and depends, as above, on fatty degeneration; and the other is a suppuration of the permanent thrombus, which results when the cellular proliferation goes beyond proper bounds. — *Glasgow Medical Journal*, *American Journal of the Medical Sciences.*

---

Termination of Nerves in the Lips. — Dr. Pallidino (*Bull. dell' Assoc. dei Natural di Napoli*) states that in the lips of the horse, which are richly supplied with nerves, many isolated, non-medullated fibres run from the subcutaneous connective tissue into the deeper layers of the epithelium, when they have a straight course and terminate by free extremities after they have traversed the deepest layer of the pavement epithelium, occasionally exhibiting a terminal dilatation or enlargement. Pallidino has not been able to discover any connection of the nerve-fibres with peculiar stellate cells of the rete malpighii, as described a year or two ago by Langerhaus.—*Lancet.*

---

Cure of a Case of Soft Malignant Tumor of the Parotid Region.—Henry Arnott, author of the valuable little monograph entitled Cancer, its varieties, its histology and diagnosis, reports a case of a soft sarcomatous growth of the parotid region which was cured by first ligating the common carotid and applying repeatedly powerful caustics. The tumor was one which it was found impossible to remove with a knife. Mr. Arnott remarks in regard to the case that:

"The case is full of encouragement for those surgeons who, seeing no chance of cutting off a cancer cleanly, prefer to let the disease alone and aim only at relieving pain. It shows how the vigorous employment of caustics can be trusted to take the place of the scalpel in situations the least promising for such destructive agents. And,

"Secondly, We may learn by such a case as this to persevere again and again with our local remedies as often as a local recurrence of the disease renders our interference necessary. Many surgeons will grapple with a primary cancer, but will refuse to meddle with the tumor which sprouts from the scar of the first operation, fearing that the return is the expression of a profound constitutional dyscrasia, which is beyond their efforts; whereas, in nine cases out of ten, it means that the first operation was not quite sweeping enough, and that a speedy attack upon the returning malady will be probably successful, unless other parts are already obviously affected."

It may be well to state that Mr. Arnott classes the softer sarcomas and the carcinomas together, under the general name of cancer.—*Am. Journal Med. Sciences.*

---

Elimination of Carbonic Oxide. —M. Grèhaut reports an experiment which, by its complexity, will not allow of more than a hasty condensation, but which completes the chain of evidence brought together by this ingenious experimenter to demonstrate that while carbonic oxide is exhaled from the lungs in ordinary conditions, it is no longer eliminated from the blood when the animal is caused to breathe confined air.—*Gaz. Hebd.*

